# Mucositis Associated with *Mycoplasma pneumoniae*: Systematic Review and Case Series

**DOI:** 10.3390/children13050638

**Published:** 2026-05-03

**Authors:** Silvia D’Agostino, Vanja Granberg, Giulia Valentini, Massimo Corsalini, Luisa Limongelli

**Affiliations:** 1 Department of Medical, Oral and Biotechnological Sciences, University G. D’Annunzio, 66100 Chieti, Italy; giulia.valentini@unich.it; 2 Department of Interdisciplinary Medicine, University of Bari “Aldo Moro”, 70124 Bari, Italy; vanjagranberg@libero.it (V.G.); massimo.corsalini@uniba.it (M.C.); luisa.limongelli@uniba.it (L.L.)

**Keywords:** Mucositis, *Mycoplasma pneumoniae*, stomatitis, conjunctivitis, MIRM

## Abstract

**Background/Objectives**: *Mycoplasma pneumoniae* (MP) is a frequent cause of community-acquired pneumonia, but it is increasingly recognized for extrapulmonary complications, specifically *Mycoplasma pneumoniae*-induced rash and mucositis (MIRM). This systematic review aims to comprehensively assess the frequency of clinical features, diagnostic criteria and outcomes of oral mucositis in patients with confirmed MP infection. **Methods**: A systematic review was conducted following PRISMA guidelines across PubMed, Web of Science and Scopus, covering the period 2015–2025. Inclusion criteria encompassed in vivo studies, case reports, and case series in English focusing on MP-associated mucositis. Methodological quality was assessed using JBI checklists for case-based evidence and the Newcastle–Ottawa Scale for cohort studies. Two clinical cases were reported. **Results**: Out of 242 identified records, 42 studies were included, involving 140 patients with a notable male predominance (62%). Oral involvement was reported in 92.9% of cases, often characterized by severe ulcerations, hemorrhagic crusting, and debilitating pain. Intensive Care Unit admission was required in 21.5% of cases due to severe systemic or mucosal disease, with 14.3% necessitating parenteral nutrition. Quality assessment indicated moderate-to-high methodological rigor across most included studies. **Conclusions**: MIRM represents a significant clinical entity where oral mucositis is a dominant feature, often preceding or overshadowing respiratory symptoms. Early recognition by oral health professionals is crucial to avoid misdiagnosis, ensure appropriate multidisciplinary care, and implement supportive or immunomodulatory therapies that reduce morbidity and hospitalization length.

## 1. Introduction

*Mycoplasma pneumoniae* (MP) is a unique, wall-less bacterium that represents one of the most frequent causes of community-acquired pneumonia (CAP) worldwide, particularly among school-aged children and young adults [1]. In endemic and epidemic settings, MP accounts for approximately 4–8% of all bacterial CAP cases, with this number increasing to 20–40% during outbreaks [2]. While its pulmonary manifestations are well recognized, MP infection is increasingly associated with a diverse array of extrapulmonary complications, affecting virtually every organ system [3]. Among these, mucocutaneous manifestations are among the most visible and diagnostically challenging, often presenting in acute care settings such as emergency departments and pediatric inpatient units [4,5].

Over the past decade, a clearer clinical and immunopathological profile has emerged for a distinct mucocutaneous condition now known as *Mycoplasma pneumoniae*-induced rash and mucositis (MIRM). Historically, patients presenting with blistering mucous membrane lesions in the setting of MP infection were misdiagnosed as having Stevens–Johnson syndrome (SJS), toxic epidermal necrolysis (TEN), or erythema multiforme major (EMM)—conditions that are more commonly drug-induced and carry significantly worse prognoses [6,7]. In 2015, Canavan et al. formally proposed MIRM as a separate clinical entity after conducting a systematic review of 202 cases, highlighting its unique presentation and favorable prognosis [8]. Since then, MIRM has been further characterized as part of the broader category of reactive infectious mucocutaneous eruptions (RIME), a term encompassing mucositis triggered by infections such as Chlamydia pneumoniae, influenza B virus, and others [3].

MIRM is now understood to follow a distinct epidemiologic and clinical pattern, with a marked predilection for children and adolescents. The average age of presentation ranges from 9 to 13 years, with a male predominance of 66–72% [5,8,9]. The disease often begins with a respiratory prodrome—including cough, fever, sore throat, and malaise—that precedes the development of mucosal lesions by an average of 7–10 days. MP infection is typically confirmed by polymerase chain reaction (PCR) from nasopharyngeal swabs or by serologic detection of IgM and IgG antibodies, though diagnostic methods vary across studies [5].

Clinically, MIRM is characterized by multifocal mucositis—most commonly affecting the oral (94–100%), ocular (82–92%), and genital (63–78%) mucous membranes [8]. Oral lesions include erosions, ulcers, vesiculobullae, hemorrhagic crusting, and diffuse sloughing of the buccal mucosa, tongue, and lips. Ocular involvement may manifest as conjunctival injection, eyelid margin ulceration, pseudomembrane formation, or—less frequently—corneal erosions, which are more common in adults than children [3,5]. Genital mucositis often presents as painful erosions and ulcerations on the labia or glans penis. Importantly, cutaneous involvement is absent or minimal in most cases: nearly one-third of MIRM cases lack any skin lesions, and when present, the rash tends to be sparse, polymorphic, and non-targetoid—unlike the lesions seen in SJS or EM [5].

The pathophysiological mechanisms underpinning MIRM are distinct from those of drug-induced SJS/TEN. Rather than being mediated by T-cell cytotoxicity and Fas-ligand expression (as in SJS/TEN), MIRM appears to be driven by innate immune activation, immune complex deposition, and molecular mimicry. The cytadherence properties of MP, particularly the P1 adhesin protein, are thought to mimic keratinocyte antigens, triggering an autoimmune response [9]. Additionally, the organism secretes toxins like hydrogen peroxide and the community-acquired respiratory distress syndrome (CARDS) toxin, which upregulate interleukin-1β (IL-1β) via inflammasome activation [10]. This leads to a cascade of proinflammatory cytokine release, local tissue damage, and mucosal inflammation. In some cases, MP DNA has been isolated directly from mucosal blister fluid, suggesting that hematogenous dissemination may also contribute to the pathology [3].

Given its distinct clinical course and immunologic profile, accurate recognition of MIRM is essential for guiding management and avoiding misclassification. Misdiagnosis as SJS/TEN may lead to inappropriate cessation of necessary medications, increased parental anxiety, and unnecessary avoidance of drugs. Unlike SJS/TEN, MIRM usually resolves without long-term sequelae, with the majority of patients recovering fully using supportive care alone—including hydration, pain control, and nutritional support. Some patients may require adjunctive treatment with antibiotics (e.g., azithromycin or doxycycline) and immunomodulatory agents such as corticosteroids or intravenous immunoglobulin (IVIG), especially in cases with extensive mucositis or ocular involvement [5,8].

Despite growing recognition, important gaps remain in our understanding of the clinical spectrum and optimal management of MIRM, particularly in terms of its oral mucosal presentation. Oral involvement is the most consistent and severe feature of MIRM, often necessitating hospitalization and multidisciplinary care involving dermatologists, pediatricians, and ophthalmologists [9,10]. This systematic review aims to comprehensively assess the presence of oral mucositis in patients with confirmed *Mycoplasma pneumoniae* infection, with a focus on the diagnostic criteria, associated clinical features, and outcomes. By synthesizing available data, this review seeks to aid clinicians in recognizing MIRM early, improving diagnostic accuracy, and guiding evidence-based treatment strategies.

## 2. Materials and Methods

A systematic review was conducted using the Preferred Reporting Items for Systematic Reviews and Meta-Analyses (PRISMA) guidelines for systematic reviews and meta-analyses [11]. While the review followed a structured internal protocol, formal registration in prospective databases was not performed for this study. All methodological steps were nevertheless fully documented to ensure transparency and reproducibility.

### 2.1. Literature Search

The primary objective of this systematic review is to synthesize current evidence on the oral manifestations of MIRM to facilitate early clinical recognition. Furthermore, this study aims to refine diagnostic accuracy within the dental setting and establish a framework for evidence-based management strategies, thereby minimizing long-term mucosal sequelae. A systematic search of PubMed, Web of Science, and Scopus was conducted, adhering to the PICO (Population, Intervention, Comparison, and Outcome) framework.

- Population: Patients diagnosed with MIRM;
- Intervention: Oral and dental diagnostic procedures and management protocols;
- Comparator: Differential clinical presentations and outcomes compared to other mucocutaneous eruptions (e.g., SJS/TEN) or different therapeutic approaches reported in the literature;
- Outcomes: Clinical resolution of oral lesions, prevention of long-term sequelae, and time to recovery.

In addition to peer-reviewed studies, the gray literature will also be considered to ensure a comprehensive synthesis of the available evidence and to minimize publication bias. As part of the supplementary search strategy, backward citation tracking (i.e., screening reference lists of included studies) will be conducted to identify potentially eligible articles not captured in the database search. The following MeSH (Medical Subject Headings) were used: (*Mycoplasma pneumoniae*); AND (mucositis).

### 2.2. Eligibility Criteria

The inclusion criteria were as follows: all in vivo studies, case reports and case series on humans analyzing the effects of *Mycoplasma pneumoniae* infection, in the English language, with a time restriction in the last ten years, 2015–2025. The search time period started on 2 May 2025 and ended on 1 September 2025.

The following served as exclusion criteria: research about mucositis due to other pathogens, patients with systemic conditions which makes the differential diagnosis not univocal; papers about the treatment of *Mycoplasma pneumoniae* infection without the case description; systematic reviews without new case reports; metanalyses; editorials; abstracts; book chapters; papers not in English. 

Studies were included based on the documented presence of oral manifestations, regardless of the depth of secondary clinical characterization.

### 2.3. Data Extraction

Studies were independently screened by two reviewers (S.D. and L.L.). In cases of disagreement, a consensus was reached through consultation with a third reviewer (G.V.). The data extraction process focused on general study characteristics (e.g., author, publication year, and funding sources) as well as specific clinical parameters, including *M. pneumoniae*-associated symptomatology, time of onset, therapeutic interventions, and follow-up data.

### 2.4. Quality Assessment

For the critical appraisal of the methodological quality and risk of bias within case reports and case series, the appropriate checklists developed by the Joanna Briggs Institute (JBI) were utilized. Specifically, the JBI Critical Appraisal Tool for Case Reports [12] was used for individual patient reports, comprising eight questions focused on criteria such as the clarity of demographic characteristics, patient history, clinical condition, diagnostic tests, intervention, and reported lessons. For studies involving multiple patients, the JBI Critical Appraisal Tool for Case Series was employed [13], which consists of ten questions assessing aspects like inclusion criteria, standard outcome measurement, follow-up, and clear reporting of patient demographics and clinical information. For both tools, each question is answered with “Yes,” “No,” “Unclear,” or “Not Applicable.” A summary score was calculated by assigning one point for every “Yes” answer, with “No,” “Unclear,” and “Not Applicable” responses scoring zero points. The final score for each study was then interpreted to determine its overall methodological quality: a higher score indicates a lower risk of bias and higher methodological quality, thus providing greater confidence in the reported findings and informing the subsequent synthesis of evidence. For practical synthesis and reporting transparency, a summary score was calculated, and studies were secondarily categorized as high (≥70% criteria met), moderate (≥50% to <70%), or low quality (<50%) based on the proportion of ‘Yes’ responses to the respective JBI Critical Appraisal Checklists. Finally, for case reports, adherence to the CARE (CAse REport) guidelines [14] was additionally considered to ensure completeness and transparency of clinical reporting. The methodological quality of included cohort studies was independently assessed using the Newcastle–Ottawa Scale (NOS) [15], which evaluates non-randomized studies across three domains: Selection, Comparability, and Outcome. Studies were awarded up to nine stars, with higher scores indicating better methodological quality. Discrepancies in scoring were resolved by consensus with the involvement of a third reviewer.

## 3. Results

### 3.1. Study Selection

**Figure 1:**
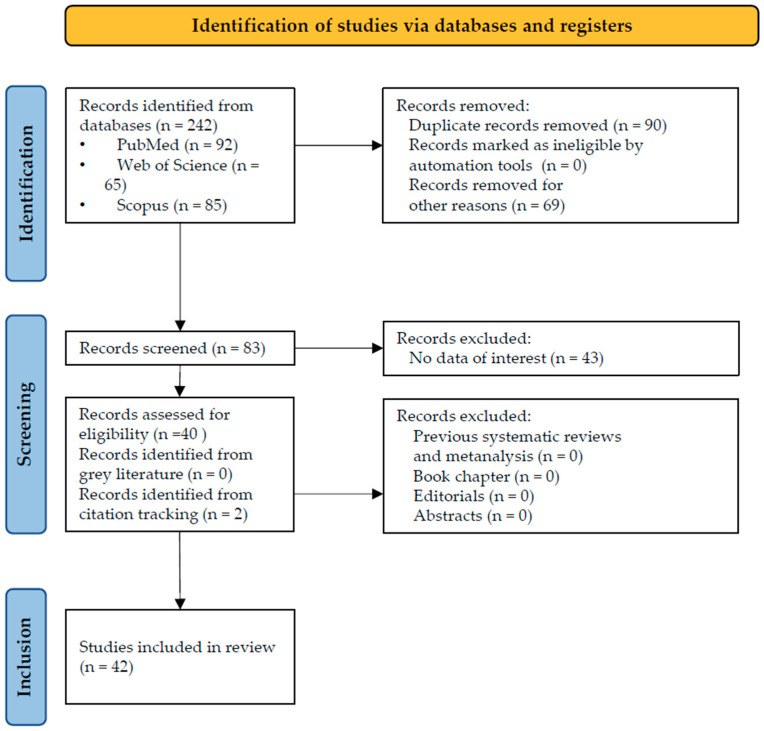
PRISMA flowchart.

The initial electronic literature search yielded a total of 242 records, distributed across the following databases: 92 from PubMed, 65 from Web of Science, and 85 from Scopus. Following the preliminary identification phase, no studies were excluded via automated screening tools; however, a total of 90 duplicate records were identified and subsequently removed to ensure the uniqueness of the dataset. Of the remaining studies, 69 were excluded after a meticulous review for failing to meet the predefined inclusion criteria. Primary reasons for exclusion included, for instance, a focus on non-target pathogens, such as *Chlamidophila pneumoniae*, *Murine tiphus*, *Streptococcus pneumoniae*, SARS-Cov-2, Influenza A, for not including mucositis, or for being about genotypes analysis, or immunological response. A total of 83 studies accessed the screening phase, and a total of 43 studies were withdrawn because they failed to demonstrate any data of interest, for example, because they had no case presentations, or explored the associations with other pathogens (e.g., SARS-Cov-2, rhinovirus), not in English, for being about Kavasaki Disease in MP patients, addressed the relationship between *M. pneumoniae* genotypes and specific clinical outcomes, explored only histological features and for being letters to the Editor. Final eligibility was confirmed for 40 studies. No records were excluded for being systematic reviews or meeting abstracts, as the inclusion focused exclusively on original research. Supplementary search strategies identified no articles from the gray literature and 2 via backward citation tracking. Consequently, 42 studies reached the final inclusion phase (Figure 1) and were subsequently analyzed in their full-text versions.

### 3.2. Detailed Results

Regarding the population age, 19% (8/42) of studies referred to children <5 y.o., and 26.2% (11/42) referred to children (5 ≤ age < 10). A total of 19% (8/42) enrolled children (10 ≤ age < 15), 16.7% (7/42) referred to adolescents (15 ≤ age < 20). A total of 19% (8/42) of studies referred to young adults aged ≥20 y.o. In total, 140 patients with reported sex were included, of whom 87 were male and 53 were female, indicating a male predominance among the reported cases. In the observational and cohort studies including multiple patients, a similar sex distribution was observed, with a slight predominance of male subjects, consistent with the overall trend identified across the included literature. Regarding the oral involvement from the study’s point of view, 92.9% (39/42) reported oral mucositis/ulcerations/stomatitis, whereas 7.1% (3/42) did not describe or did not explicitly report oral mucosal involvement. The discharge within ≤7 days was reported by 33.3% (14/42) of included studies, hospitalization >7 days was described in 26.2% (11/42) of studies. Finally, the length of stay was not explicitly reported in 40.5% (17/42) of studies. Intensive Care Unit (ICU) admission was consistently associated with severe systemic involvement and extensive mucosal disease. ICU admission was reported by 21.5% (9/42) of studies, while 78.5% (33/42) declared no ICU admission. Parenteral nutrition was explicitly reported in 14.3% (6/42) of studies, while it was not shown in 85.7% (36/42) of studies. In all reported cases, parenteral nutrition was required due to severe oral mucositis with inability to maintain adequate oral intake.

The totality of the included study was proposed in Table A1 including authors, year, study design, sex and age, mucositis onset and treatment of the investigated subjects.

### 3.3. Quality Assessment Results

The methodological quality of the 39 included studies (including both case reports and case series) was assessed according to JBI Critical Appraisal Checklists. Regarding case reports (n = 29), the majority of the evidence demonstrated high reporting quality. Specifically, 62.1% (18/29) of the studies achieved the maximum JBI score of 8/8. Furthermore, 24.1% (7/29) met seven out of eight criteria, while the remaining 13.8% (4/29) met six criteria. Common areas of uncertainty or lack of reporting (responses ‘U’ or ‘N’) primarily concerned the description of the patient’s clinical condition or the clarity of the follow-up details. Regarding the case series (n = 10), the quality assessment for the case series also showed robust results. In total, 40% (4/10) of the series fulfilled all 10 JBI criteria. The remaining 60% (6/10) achieved scores between eight and nine affirmative responses, with the most frequent limitations involving the description of the demographic characteristics of the series or the completeness of the statistical analysis of the clinical results. Overall, the methodological quality of the synthesized evidence can be considered high, with 100% of the studies meeting at least 75% of their respective quality criteria. This confirms the internal validity and clinical reliability of the reported oral manifestations of MIRM across the selected literature. Meanwhile, all three cohort studies demonstrated good methodological quality according to the NOS. Although these tools have intrinsic limitations, particularly when applied to predominantly descriptive and heterogeneous evidence and no single instrument is specifically tailored to rare conditions largely documented through case-based literature, they represent the most appropriate and methodologically accepted approach currently available for quality assessment in this context.

The results of the methodological quality assessment for the included studies are summarized in Table A2, Table A3 and Table A4.

### 3.4. Case Presentation

#### 3.4.1. Case 1

**Figure 2:**
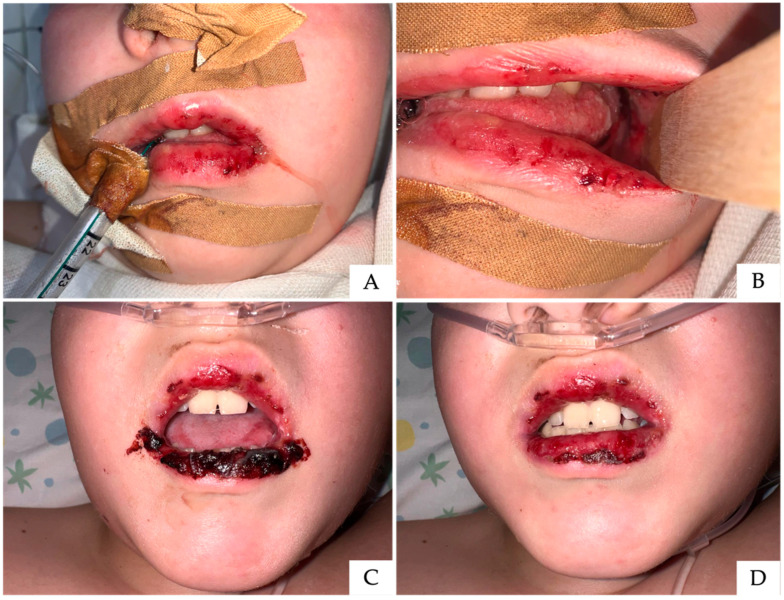
Acute phase of the first case. Owing to respiratory deterioration, the patient requires endotracheal intubation (**A**) and presents severe stomatitis affecting the oral mucosa (**B**). Severe involvement of the lips is evident, characterized by extensive ulcerations and hemorrhagic crusting (**C**,**D**).

A previously healthy 10-year-old male presented with a history of high-grade fever (38–39 °C), and was initially managed at home with water-soluble ibuprofen (twice daily) and paracetamol as needed. Due to the persistence of fever after four days, the patient started a cycle of amoxicillin/clavulanic acid (one tablet daily for two days). On the fifth day, oral vesicles appeared and were treated topically with an aphthous gel while continuing ibuprofen. By the sixth day, the patient was evaluated by a pediatrician. Clinical examination revealed no lymphadenopathy. Based on a clinical suspicion of herpetic stomatitis, the patient was prescribed oral acyclovir (5 mL every 5 h). Both the antibiotic and ibuprofen were discontinued, and aerosol therapy was initiated. On the seventh day, the patient was referred to an oral pathologist. He presented with severe, debilitating pain and an inability to maintain oral hydration; acyclovir was discontinued, and a single 8 mg dose of dexamethasone was administered as recommended by the oral pathologist. By the eighth day, the clinical status deteriorated significantly, prompting emergency department admission. On presentation, the patient appeared somnolent and lethargic, with a grayish skin pallor. Physical examination revealed extensive vesiculation, desquamation, and spontaneous bleeding of the oral mucosa. The patient was immediately intubated and transferred to the pediatric Intensive Care Unit for acute respiratory failure (Figure 2A,B), with an oxygen saturation of 82% and a diagnosis of bilateral pneumonia. Microbiological investigations identified *Mycoplasma pneumoniae* infection via PCR and specific serology. Intravenous clarithromycin was initiated at a dose of 15 mg/kg/day, administered in two divided doses. Following clinical stabilization, vital parameters improved markedly, allowing extubation and transfer to the Infectious Diseases unit. Crusted lip lesions (Figure 2C,D) were gently removed at least twice daily using gauze soaked in hydrogen peroxide, followed by application of glycerin-based emollient and protective creams. At completion of a 12-day course of antibiotic therapy, the pulmonary findings showed significant resolution, and the patient was discharged with home-based treatment. Home-based topical management consisted of application of a hyaluronic acid–based gel until complete clinical resolution. The patient subsequently underwent follow-up with his dentist and did not require further consultation at our center.

#### 3.4.2. Case 2

**Figure 3:**
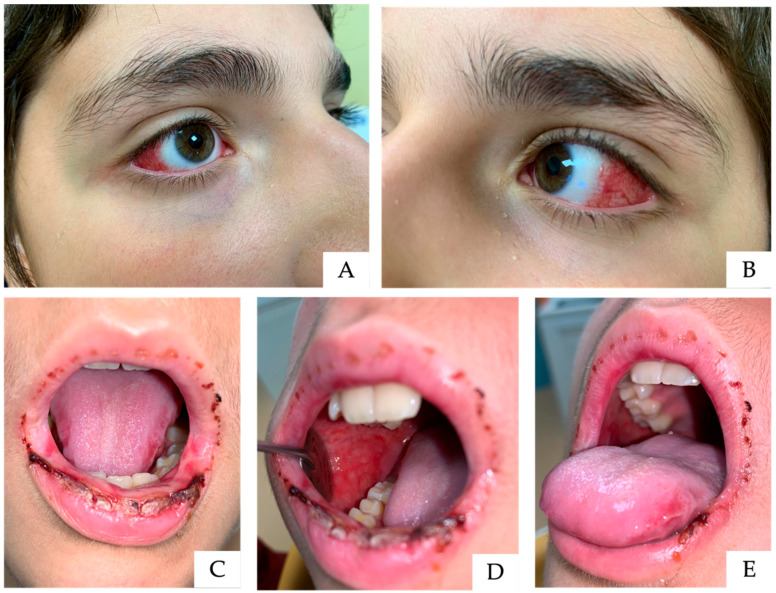
Acute phase of the second case, showing marked bilateral conjunctival involvement (**A**,**B**). The lips exhibit extensive ulceration with overlying crusts (**C**–**E**), and additional mucosal lesions are visible along the lateral margin of the tongue, consistent with widespread oral involvement (**D**).

**Figure 4:**
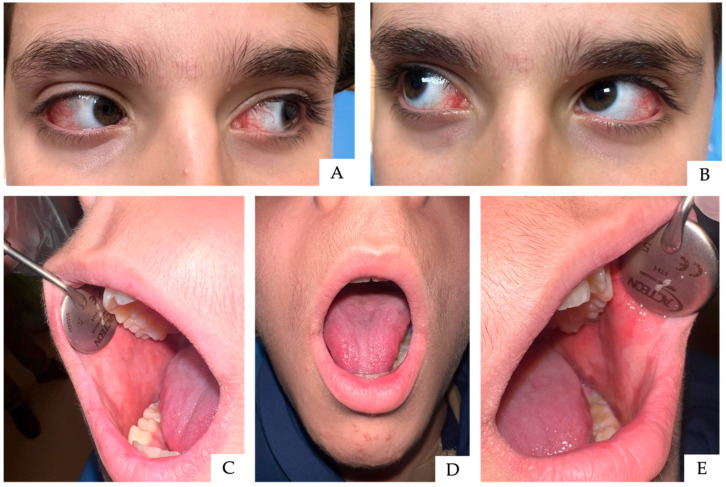
Clinical resolution. Healing of conjunctival involvement was achieved (**A**,**B**). Healing of the lips (**C**–**E**).

An 11-year-old male patient was admitted with a PCR and serology diagnosis of *Mycoplasma pneumoniae* pneumonia and was treated with intravenous clarithromycin at a dosage of 15 mg/kg/day, administered in two divided doses. The patient was referred to the Pediatric Dentistry Unit because of significant feeding difficulties secondary to severe oral and perioral burning sensations. Clinical examination revealed diffuse ulcerative and crusted lesions involving the lips, along with extensive lesions affecting the lateral borders of the tongue and the entire buccal mucosa (Figure 3C–E). Additionally, marked marginal conjunctival hyperemia was observed (Figure 3A,B). The patient also exhibited vesiculobullous and ulcerative lesions of the glans penis assessed by a dermatologist; however, photographic documentation was not permitted. Mouth rinses containing a low concentration of chlorhexidine (0.05%) were recommended and administered as part of the supportive management. A hyaluronic acid-based gel combined with an aminoacidic complex was applied until complete healing of the oral mucosa was achieved, as documented in Figure 4. Follow-up examination revealed a complete resolution of genital lesions, with no evidence of long-term sequelae.

## 4. Discussion

This systematic review synthesizes available evidence on oral mucositis associated with *Mycoplasma pneumoniae* infection, with particular attention to the entity currently referred to as *Mycoplasma pneumoniae*-induced rash and mucositis (MIRM). Across the 42 included studies, oral mucosal involvement emerged as a central and often dominant clinical feature, frequently representing the primary cause of morbidity and the main driver for hospital admission. Although initially described within the dermatological and pediatric literature, the findings of this review underscore the critical relevance of MIRM for oral health professionals, given the severity, extent, and functional consequences of oral lesions.

### 4.1. Clinical Phenotype and Predominance of Oral Involvement

A consistent observation across case reports, case series, and cohort studies is the prominence of oral mucositis, often severe and disproportionate compared with cutaneous involvement. Many studies describe extensive ulcerations of the lips, buccal mucosa, tongue, palate, and oropharynx, frequently accompanied by hemorrhagic crusting and intense pain that significantly impairs oral intake, speech, and oral hygiene [16,17,18]. In several reports, skin involvement was minimal or absent, involving less than 10% of body surface area, reinforcing the concept that MIRM differs from classic Stevens–Johnson syndrome (SJS) and erythema multiforme (EM) [19,20]. Importantly, oral mucositis was frequently the earliest or most striking manifestation, occasionally preceding pulmonary findings or occurring in the context of mild or initially unrecognized respiratory symptoms [21,22]. This temporal pattern has significant diagnostic implications for dental practitioners, who may be the first clinicians to evaluate these patients. Several studies emphasize that isolated or predominant oral involvement can lead to misdiagnosis as primary herpetic gingivostomatitis, aphthous stomatitis, or drug-induced mucositis, resulting in delays in appropriate management [23,24].

### 4.2. Age Distribution and Sex Differences

The reviewed literature confirms a marked predominance of pediatric and adolescent patients, with most cases occurring in children between 4 and 15 years of age. Cohort and case series data indicate that mucocutaneous manifestations are significantly more frequent in children with *M. pneumoniae*-related community-acquired pneumonia compared with other etiologies [25]. Nonetheless, multiple well-documented adult cases demonstrate that MIRM is not confined to the pediatric population, with young and middle-aged adults presenting similar oral and mucosal phenotypes [26,27,28].

A male predominance was observed across most studies, particularly in pediatric cohorts and larger case series [29,30]. While the biological basis for this sex difference remains unclear, it appears consistent across geographic regions and study designs. From an oral medicine perspective, this demographic profile may inform clinical suspicion when evaluating acute, severe oral mucositis in male pediatric patients with recent respiratory symptoms.

### 4.3. Pathophysiological Considerations

Although the precise mechanisms underlying MIRM remain incompletely understood, the reviewed studies collectively support an immune-mediated pathogenesis rather than direct microbial invasion of the mucosa. The typical delay of several days between respiratory symptoms and mucosal involvement, along with elevated inflammatory markers and favorable responses to immunomodulatory therapy, suggests a post-infectious inflammatory process [25,29]. This hypothesis is further supported by recurrent cases and familial clustering, as reported by Song et al. [31], indicating a possible host susceptibility or genetic predisposition. For oral tissues, this immune-driven damage results in widespread epithelial disruption, ulceration, and secondary inflammation, which may predispose to superinfection, dehydration, and long-term sequelae such as labial adhesions or scarring if not managed appropriately [24,32]. These findings reinforce the need for early recognition and multidisciplinary management, including dental and oral medicine input. To prevent long-term sequelae like labial adhesions and microstomia, the dentist must implement frequent atraumatic debridement and apply high-potency topical corticosteroids. Maintaining a protective lipid barrier (e.g., sterile petrolatum) is critical to prevent the apposition of raw mucosal surfaces, while gentle, passive range-of-motion exercises should be supervised to preserve tissue elasticity and prevent cicatricial contraction.

### 4.4. Therapeutic Approaches and Implications for Oral Care

No standardized treatment guidelines for MIRM currently exist, and management strategies varied widely across studies. Antibiotic therapy targeting *M. pneumoniae*, most commonly macrolides or tetracyclines, was universally employed and remains the cornerstone for treating the underlying infection. However, antibiotics alone were often insufficient to control mucosal inflammation, particularly in severe oral involvement [16,23]. Systemic corticosteroids were frequently used, especially in patients with extensive oral mucositis, with many reports describing rapid improvement in pain, oral intake, and lesion resolution following steroid initiation [17,33]. Adjunctive therapies such as intravenous immunoglobulin (IVIG) and cyclosporine A were reserved for severe or refractory cases, with some evidence suggesting benefit when administered early [16,34]. However, the heterogeneity of treatment regimens and lack of comparative studies preclude definitive conclusions regarding optimal therapy. From a dental clinical standpoint, local oral management was inconsistently reported but appears crucial. Supportive measures including analgesic mouthwashes, topical corticosteroids, emollients, meticulous oral hygiene, and prevention of labial adhesions were highlighted in several studies as key components of care [32,35]. Failure to address oral-specific needs may contribute to prolonged morbidity, nutritional compromise, and long-term functional impairment.

### 4.5. Differential Diagnosis and Diagnostic Challenges

A recurrent theme across the literature is the diagnostic overlap between MIRM, SJS, EM, and drug-induced mucositis. Many patients were initially misclassified, particularly when oral lesions were severe and skin findings minimal [36,37]. Distinguishing features favoring MIRM include predominant mucositis involving multiple mucosal sites, sparse cutaneous lesions, a history of recent respiratory infection, and laboratory evidence of *M. pneumoniae* infection.

For dental clinicians, awareness of MIRM is particularly important, as early oral findings may precede dermatological consultation or systemic diagnosis. Prompt recognition can facilitate appropriate referral, microbiological testing, and avoidance of unnecessary drug discontinuation or misattribution to allergic reactions.

### 4.6. Limitations of the Study

This review is limited by the nature of the available evidence, which consists predominantly of case reports and small case series, with only a few retrospective and cohort studies. The heterogeneity in diagnostic criteria, terminology (MIRM, MPAM, atypical SJS), and treatment protocols limits comparability across studies. Additionally, oral findings were variably described, and standardized oral outcome measures were lacking. Publication bias toward severe or atypical cases is likely, potentially overestimating disease severity and intervention intensity. Furthermore, this review did not evaluate the comparative sensitivity or specificity of diagnostic methods (e.g., PCR versus serology), as the primary focus was restricted to the clinical characterization and dental management of oral manifestations.

### 4.7. Clinical Relevance

For dental and oral medicine practitioners, MIRM represents a clinically significant but underrecognized cause of acute, severe oral mucositis, particularly in pediatric patients. Early identification of characteristic oral lesions in the context of recent respiratory symptoms can expedite diagnosis, improve multidisciplinary management, and reduce morbidity. Dentists play a critical role in pain control, maintenance of oral function, prevention of complications, and long-term follow-up, underscoring the importance of incorporating MIRM into the differential diagnosis of acute ulcerative oral diseases.

## 5. Conclusions

In conclusion, this systematic review and case series demonstrate that oral mucositis is the hallmark manifestation of MIRM. The evidence confirms a high prevalence among male children and adolescents, characterized by severe, painful ulcerations and hemorrhagic crusting. The analyzed literature and the two clinical cases presented—ranging from multi-organ mucosal involvement to severe respiratory failure—highlight the critical nature of the acute phase and the frequent need for intensive supportive care. These findings emphasize that early clinical recognition of oral markers by healthcare professionals is essential to ensure a correct diagnosis and a prompt, multidisciplinary therapeutic approach.

## Data Availability

All datasets generated during this study are included within the manuscript. Additional data are available from the corresponding author upon reasonable request; original clinical photographs and primary records for the institutional cases are archived and available from Luisa Limongelli.

## Appendix A. Detailed Search Strategy and Reproducibility

Search Period: 2 May 2025–1 September 2025.
Time Restriction: 2015–2025 (Primary Database Search).
Language: English.
Population: Humans (In vivo studies).

To ensure full reproducibility and address the requirement for a broader identification of the MIRM-related literature (including synonyms such as RIME and MPAM), the following search strings were utilized:

1. *PubMed/MEDLINE*

(“Mycoplasma pneumoniae” [Mesh] OR “Mycoplasma pneumoniae” [Title/Abstract]) AND (“mucositis”[Title/Abstract] OR “stomatitis”[Title/Abstract] OR “oral manifestations” [Title/Abstract] OR “MIRM” [Title/Abstract] OR “RIME” [Title/Abstract] OR “reactive infectious mucocutaneous eruption” [Title/Abstract] OR “atypical Stevens-Johnson syndrome” [Title/Abstract] OR “MPAM” [Title/Abstract])

Filters applied: Humans, English, Publication date from 2 May 2015 to 1 September 2025.

2. *Scopus*

TITLE-ABS-KEY (“Mycoplasma pneumoniae” AND (“mucositis” OR “stomatitis” OR “oral manifestations” OR “MIRM” OR “RIME” OR “reactive infectious mucocutaneous eruption” OR “atypical Stevens-Johnson syndrome” OR “MPAM”))

Filters applied: language (English), date range (2015–2025).

3. *Web of Science (Core Collection)*

TS = (“Mycoplasma pneumoniae”) AND TS = (“mucositis” OR “stomatitis” OR “oral manifestations” OR “MIRM” OR “RIME” OR “reactive infectious mucocutaneous eruption” OR “atypical Stevens-Johnson syndrome” OR “MPAM”)

Timespan: 2015–2025. Language: English.

**Table A1:** Main results. Age is expressed by mean value, absolute or range. Y.o., years old. IVIG, intravenous immunoglobulin.

Authors/Years	Study Design	Sex/Age (y.o., Mean or Pure)	Mucositis Onset	Treatment
Cool D. et al. 2025 [26]	Case series	75% M, 25% F/11.2	Oral and ocular mucosal involvement was present in all patients, with urogenital mucositis reported in 50% of cases.	No specific oral treatment was described; ocular involvement was managed with preservative-free steroids, lubricants, and topical antibiotics.
Silva KRPD. et al. 2025 [38]	Case report	M/4	Disseminated lesions involved the oral and genital mucosa with acral predominance.	Supportive care, systemic clarithromycin, short-course systemic corticosteroids, topical emollients, laser therapy for oral lesions, and ophthalmic antibiotics with steroids.
Yoosuf FT. et al. 2025 [39]	Case report	M/25	Painful oral lesions and ocular involvement developed two days after respiratory symptom onset.	Intravenous fluids, IVIG, systemic corticosteroids, cyclosporine, antibiotics, and topical emollients.
Hasbini J. et al. 2024 [40]	Case report	F/7	Aphthous stomatitis and mucositis developed following several days of fever and conjunctivitis.	Specific treatment for oral mucositis was not reported.
Kucharek I. et al. 2024 [41]	Case report	F/7	Stomatitis and conjunctivitis preceded respiratory deterioration and salivary gland inflammation.	Systemic antibiotics, systemic corticosteroids, antifungals for oral lesions, antiviral therapy and ophthalmologic interventions.
Li C. et al. 2024 [42]	Case report	M/6	Severe oral mucosal damage occurred in association with fever, cough, and cutaneous rash.	Supportive care, macrolide therapy, ceftriaxone, and systemic corticosteroids were administered.
Wang P. et al. 2024 [43]	Retrospective analysis	43% M, 57% F/5.74	Oral mucositis was not specifically reported in this cohort.	Intravenous methylprednisolone and symptomatic therapy, with bronchoalveolar lavage in selected cases.
Zhang X. et al. 2024 [44]	Retrospective analysis	54% M, 46% F/<18	Mucocutaneous involvement was not specifically detailed.	Conventional antibiotics, anticoagulation, thrombolysis or thrombectomy when indicated and advanced respiratory support.
Lu H. et al. 2023 [45]	Case report	F/8	Painful oral ulcers developed several days after the onset of fever and cough.	Supportive care and doxycycline were administered, with spontaneous resolution of mucocutaneous lesions.
Beheshti R. et al. 2022 [33]	Case report	M/10	Oral mucositis and conjunctivitis developed early during a febrile respiratory illness.	Intravenous corticosteroids and azithromycin led to rapid resolution of mucositis.
Ben Rejeb M. et al. 2022 [19]	Case series	F, M/13.5	Severe ulcerative oral mucositis developed shortly after respiratory symptoms and conjunctivitis.	Systemic corticosteroids and antibiotics were used, with topical corticosteroids for oral and ocular lesions.
Chen N. et al. 2022 [29]	Case series	80% M, 20% F/7 (3 < 2 y.o.)	Mucocutaneous lesions appeared 2–11 days after respiratory symptoms.	Macrolides, systemic corticosteroids, and IVIG were commonly administered, with prolonged treatment durations in some cases.
Mosca S. et al. 2022 [21]	Case report	F, M/16	Oral ulcerations developed after several days of fever and upper respiratory symptoms.	Azithromycin and supportive care were provided, with intravenous fluids and topical treatments in more severe cases.
Slauer RD. et al. 2022 [46]	Case report	Trans M/19	Oral ulcerations were present at admission and preceded severe systemic involvement.	Broad-spectrum antibiotics and supportive management were administered.
Woodhull S. et al. 2022 [23]	Case report	M/12	Painful oral blisters developed early during a febrile respiratory illness.	Initial antiviral therapy was followed by antibiotics and systemic corticosteroids, resulting in rapid improvement.
Yadava SK. et al. 2022 [47]	Case report	M/43	Oral mucosal bleeding and lesions followed several days of fever and cough.	Antibiotics with systemic corticosteroids considered for immune-mediated manifestations.
Carvalho AA. et al. 2021 [35]	Case report	M/4	Progressive oral lesions developed in the context of worsening respiratory symptoms.	Multimodal therapy included antibiotics, systemic corticosteroids, IVIG, surgical debridement, and supportive oral care.
Go JR. et al. 2021 [27]	Case report	M/38	Oral ulcerations occurred alongside respiratory symptoms and conjunctival involvement.	Azithromycin and systemic corticosteroids resulted in complete resolution.
Maredia H. et al. 2021 [48]	Case series	2F, 1M/14.3	Recurrent episodes of oral mucositis occurred with variable mucosal involvement.	Antibiotics and systemic corticosteroids were consistently used.
Sheth HS. et al. 2021 [49]	Case series	M/22.5	Oral lesions developed within days of respiratory and systemic symptoms.	Antibiotics, systemic corticosteroids, topical oral agents, and supportive care were administered.
Thangaraju S. et al. 2021 [50]	Case report	F/6	Hemorrhagic oral crusting developed several days after respiratory symptoms.	Azithromycin, systemic corticosteroids, and topical ophthalmic and oral therapies were effective.
Valle J. et al. 2021 [24]	Case report	M/9	Oral ulcerations appeared early and progressively worsened during hospitalization.	Antibiotic therapy, nutritional support, and prolonged inpatient care were required.
Burns EK. et al. 2020 [51]	Case report	M/19	Painful oral blistering occurred concurrently with fever and conjunctivitis.	Fluoroquinolone therapy and extensive supportive and topical care were provided.
Chowdhury SR. et al. 2020 [20]	Case report	M/6	Severe oral mucositis followed several days of fever and respiratory symptoms.	Clarithromycin therapy led to rapid defervescence and gradual mucosal recovery.
Jin HD. et al. 2020 [52]	Case report	F/13	Oral ulcers developed after several days of fever and cough.	Topical ocular corticosteroids and antibiotics.
Lambert T et al. 2020 [53]	Case report	M/18	Oral lesions developed after initial genital and ocular involvement.	Antibiotics, antiviral therapy, and systemic corticosteroids were administered.
Liu LP et al. 2020 [54]	Case series	3M, 2F/5.16	All patients developed oral ulcerations with ocular and genital involvement.	Azithromycin and systemic corticosteroids were used.
Lofgren DH. et al. 2020 [55]	Case report	M/24	Oral, ocular, and genital mucositis developed after prolonged respiratory symptoms.	Supportive care combined with intravenous azithromycin and ceftriaxone led to improvement.
Meyer Sauteur PM. et al. 2020 [25]	Cohort study	55%M, 45%F/5.7	Oral ulcerations occurred in a subset of children with *M. pneumoniae*–associated pneumonia.	Antibiotics targeting *M. pneumoniae* and short-course systemic corticosteroids were administered.
Rollins PD. et al. 2020 [16]	Case report	M/9	Severe, diffuse oral ulceration developed alongside multisite mucosal involvement.	Systemic corticosteroids, antibiotics, nutritional support, and intensive local care were required.
Li HO. et al. 2019 [34]	Case series	33% M, 66%F/10.3	Oral and genital mucosal lesions developed after respiratory symptoms.	Antibiotics, supportive care, and cyclosporine A were used with favorable outcomes.
Amode R. et al. 2018 [30]	Case series	64%M, 36% F/32.3	Oral involvement was not reported in this cohort.	Macrolides and systemic corticosteroids were commonly used.
Ashton R. 2018 [32]	Case report	F/6	Severe oral mucositis developed after prolonged upper respiratory infection.	Antibiotics and supportive care were provided, with surgical intervention required for sequelae.
Curtiss P. et al. 2018 [56]	Case report	M/15	Progressive oral mucositis developed following respiratory and systemic symptoms.	Azithromycin and supportive care were administered.
Song H. et al. 2018 [31]	Case series	M/28.5	Oral and genital mucosal erosions developed after upper respiratory infection.	Supportive care, azithromycin, and IVIG were used in recurrent cases.
Bukhari EE. et al. 2017 [22]	Case report	M/12	Oral mucositis developed several days after fever and antibiotic initiation.	Clarithromycin and supportive oral and ocular care were administered.
Poddighe D. et al. 2017 [17]	Case report	M/10	Severe oral mucosal lesions developed following a self-limiting respiratory illness.	Intravenous hydration, systemic corticosteroids, and clarithromycin led to remission.
Alcántara-Reifs CM. et al. 2016 [28]	Case report	M/35	Oral ulcerations developed concurrently with fever and conjunctivitis.	Intravenous corticosteroids resulted in rapid symptom resolution.
Winikor JM. et al. 2016 [36]	Case report	M/14	Oral and genital mucositis developed days after upper respiratory symptoms.	Antibiotics and systemic corticosteroids were administered.
Vujic I. 2015 [37]	Case report	M/23	Painful oral ulcerations developed acutely after fever and cough.	Doxycycline and high-dose systemic corticosteroids led to rapid recovery.
Hillebrand-Haverkort ME. et al. 2008 [57]	Case report	M/23	Oral ulcerations developed approximately one week after symptom onset.	Amoxicillin–clavulanate followed by azithromycin was administered.
Ravin KA. et al. 2007 [18]	Case series	M/13.7	Oral mucositis developed several days after fever and respiratory symptoms in all cases.	Antibiotics, systemic corticosteroids, supportive care, and nutritional support.

**Table A2:** Methodological quality assessment of cohort studies using the JBI critical appraisal checklist for case reports. Y, yes. N, no. U, unclear.

Authors/Years	1	2	3	4	5	6	7	8
Silva KRPD. et al. 2025 [38]	Y	Y	Y	Y	Y	Y	U	Y
Yoosuf FT. et al. 2025 [39]	Y	Y	Y	Y	Y	Y	Y	Y
Hasbini J. et al. 2024 [40]	Y	Y	Y	Y	Y	U	Y	Y
Kucharek I. et al. 2024 [41]	Y	Y	Y	Y	U	Y	Y	Y
Li C. et al. 2024 [42]	Y	Y	Y	Y	Y	Y	Y	Y
Lu H. et al. 2023 [45]	Y	Y	Y	Y	Y	Y	Y	Y
Beheshti R. et al. 2022 [33]	Y	Y	Y	Y	Y	N	Y	Y
Mosca S. et al. 2022 [21]	Y	Y	Y	Y	U	Y	Y	Y
Slauer RD. et al. 2022 [46]	Y	Y	Y	Y	Y	Y	N	Y
Woodhull S. et al. 2022 [23]	Y	Y	Y	Y	N	Y	Y	Y
Yadava SK. et al. 2022 [47]	Y	Y	Y	Y	Y	Y	Y	Y
Carvalho AA. et al. 2021 [35]	Y	Y	Y	Y	Y	U	Y	Y
Go JR. et al. 2021 [27]	Y	Y	Y	Y	Y	Y	Y	Y
Thangaraju S. et al. 2021 [50]	Y	Y	Y	Y	Y	Y	U	Y
Valle J. et al. 2021 [24]	Y	Y	Y	Y	N	Y	Y	Y
Burns EK. et al. 2020 [51]	Y	Y	Y	Y	Y	Y	Y	Y
Chowdhury SR. et al. 2020 [20]	Y	Y	Y	Y	Y	Y	Y	Y
Jin HD. et al. 2020 [52]	Y	Y	Y	Y	N	Y	U	Y
Lambert T et al. 2020 [53]	Y	Y	Y	Y	Y	Y	Y	Y
Lofgren DH. et al. 2020 [55]	Y	Y	Y	Y	Y	U	Y	Y
Rollins PD. et al. 2020 [16]	Y	Y	Y	Y	Y	Y	Y	Y
Ashton R. 2018 [32]	Y	Y	Y	Y	Y	Y	Y	Y
Curtiss P. et al. 2018 [56]	Y	Y	Y	Y	Y	N	Y	Y
Bukhari EE. et al. 2017 [22]	Y	Y	Y	Y	Y	Y	Y	Y
Poddighe D. et al. 2017 [17]	Y	Y	Y	Y	Y	Y	Y	Y
Alcántara-Reifs CM. et al. 2016 [28]	Y	Y	Y	Y	Y	Y	Y	Y
Winikor JM. et al. 2016 [36]	Y	Y	Y	Y	Y	Y	Y	Y
Vujic I. 2015 [37]	Y	Y	Y	Y	Y	Y	Y	Y
Hillebrand-Haverkort ME. et al. 2008 [57]	Y	Y	Y	Y	Y	Y	Y	Y

**Table A3:** Methodological quality assessment of cohort studies using the JBI critical appraisal checklist for case series. Y, yes. N, no. U, unclear.

Authors/Years	1	2	3	4	5	6	7	8	9	10
Cool D. et al. 2025 [26]	Y	Y	Y	Y	U	Y	N	Y	Y	Y
Ben Rejeb M. et al. 2022 [19]	Y	Y	Y	Y	Y	Y	Y	Y	Y	Y
Chen N. et al. 2022 [29]	Y	Y	Y	Y	Y	N	Y	Y	N	Y
Maredia H. et al. 2021 [48]	Y	Y	Y	Y	Y	Y	Y	Y	Y	Y
Sheth HS. et al. 2021 [49]	Y	Y	Y	Y	N	Y	Y	Y	Y	Y
Liu LP et al. 2020 [54]	Y	Y	Y	Y	Y	Y	Y	Y	Y	Y
Li HO. et al. 2019 [34]	Y	Y	Y	Y	Y	Y	N	Y	Y	Y
Amode R. et al. 2018 [30]	Y	Y	Y	Y	N	Y	Y	Y	Y	Y
Song H. et al. 2018 [31]	Y	Y	Y	Y	Y	Y	Y	Y	U	Y
Ravin KA. et al. 2007 [18]	Y	Y	Y	Y	N	Y	Y	Y	Y	Y

**Table A4:** Methodological quality assessment of cohort studies using the Newcastle–Ottawa Scale (NOS).

Authors/Years	Selection (0–4)	Comparability (0–2)	Outcome (0–3)	Total Score (0–9)
Wang P. et al. 2024 [43]	★★★★	★★	★★★	9
Zhang X. et al. 2024 [44]	★★★★	★★	★★★	9
Meyer Sauteur PM. et al. 2020 [25]	★★★	★★	★★★	8
